# A machine learning pipeline for quantitative phenotype prediction from genotype data

**DOI:** 10.1186/1471-2105-11-S8-S3

**Published:** 2010-10-26

**Authors:** Giorgio Guzzetta, Giuseppe Jurman, Cesare Furlanello

**Affiliations:** 1 Fondazione Bruno Kessler, Trento, Italy; 2 DISI, University of Trento, Trento, Italy

## Abstract

**Background:**

Quantitative phenotypes emerge everywhere in systems biology and biomedicine due to a direct interest for quantitative traits, or to high individual variability that makes hard or impossible to classify samples into distinct categories, often the case with complex common diseases. Machine learning approaches to genotype-phenotype mapping may significantly improve Genome-Wide Association Studies (GWAS) results by explicitly focusing on predictivity and optimal feature selection in a multivariate setting. It is however essential that stringent and well documented Data Analysis Protocols (DAP) are used to control sources of variability and ensure reproducibility of results. We present a genome-to-phenotype pipeline of machine learning modules for quantitative phenotype prediction. The pipeline can be applied for the direct use of whole-genome information in functional studies. As a realistic example, the problem of fitting complex phenotypic traits in heterogeneous stock mice from single nucleotide polymorphims (SNPs) is here considered.

**Methods:**

The core element in the pipeline is the L1L2 regularization method based on the naïve elastic net. The method gives at the same time a regression model and a dimensionality reduction procedure suitable for correlated features. Model and SNP markers are selected through a DAP originally developed in the MAQC-II collaborative initiative of the U.S. FDA for the identification of clinical biomarkers from microarray data. The L1L2 approach is compared with standard Support Vector Regression (SVR) and with Recursive Jump Monte Carlo Markov Chain (MCMC). Algebraic indicators of stability of partial lists are used for model selection; the final panel of markers is obtained by a procedure at the chromosome scale, termed ’saturation’, to recover SNPs in Linkage Disequilibrium with those selected.

**Results:**

With respect to both MCMC and SVR, comparable accuracies are obtained by the L1L2 pipeline. Good agreement is also found between SNPs selected by the L1L2 algorithms and candidate loci previously identified by a standard GWAS. The combination of L1L2-based feature selection with a saturation procedure tackles the issue of neglecting highly correlated features that affects many feature selection algorithms.

**Conclusions:**

The L1L2 pipeline has proven effective in terms of marker selection and prediction accuracy. This study indicates that machine learning techniques may support quantitative phenotype prediction, provided that adequate DAPs are employed to control bias in model selection.

## Background

Fitting quantitative phenotypes from genome-wide data is a rapidly emerging research area, also object of dedicated data contests [1-3]. Given the complexity of the molecular mechanisms underlying many common human diseases, one of the most significant challenges to catch genetic variations associated to functional effects is enabling a modeling approach that is really multivariate and predictive [4]. In particular, it is clear that modeling should be based on patterns of multiple SNPs (with patterns’ structure extending the notion of haplotype) rather than on single SNPs. Attention is thus directed towards machine learning methods that can provide SNP selection simultaneously with the regression model, and manage high-order interactions and correlation effects among features. In this view, a handy off-the-shelf solution is the application of the Random Forest method [5], available with fast implementations (e.g. RandomJungle: http://www.randomjungle.org) both for classification (case-control studies) or regression (quantitative phenotype fitting). Regarding the haplotype data pattern problem, new kernel functions have been proposed for predictive classification by Support Vector Machines (SVM) in a cross-validation experimental framework [6].

Given that flexible machine learning methods for genotype data are becoming available, the second top challenge is building around the modeling exercise a framework that controls the sources of variability involved in the process. Lack of reproducibility in GWAS has been investigated and is known to have multiple causes [7]. Some of the technical causes may well transfer to genotype analyses by multivariate machine learning. Specifically, it is critical to consider the risk of selection bias [8,9] to warrant that predictive values and molecular markers be reproducible across studies on massive genotype datasets. The issue of reproducibility regards the whole sequence of preparatory and preprocessing steps (upstream analysis), model selection, application and validation (downstream analysis).

Baggerly and Coombes [10] proposed a “forensic bioinformatics” approach to revise a highly-influential series of medical papers on genomic signatures predicting response to chemotherapeutic agents. Their attempt at reproduction of the original results led to the discovery of a series of fatal flaws on data preparation and application of methods to publicly-available microarray and preclinical chemo-sensitivity data for several cancer cell lines. A series of clinical trials has been suspended as a consequence. For machine learning methods, the stage of model selection is usually the most complex. To overcome variability and bias effects arising from choices hidden in the modeling path, a serious effort has been provided by the FDA’s led initiatives MAQC and MAQC-II [11]. In particular, for classifiers of microarray data, the MAQC-II consortium has studied how predictivity and stability of biomarkers is associated to the type of adopted Data Analysis Protocol (DAP), intended as a standardized description of all steps in training, model selection and validation on novel data [12]. The type of internal and external validation methods used for selection of the best markers and models results as one of the main effects on predictive accuracy. Interactive effects of choices in the analysis design (e.g. batch size and composition) have been demonstrated also in GWAS in an extension of the MAQC-II study [13]. However, limited efforts have been directed to detailed DAPs in the regression framework, and on genotype data in particular. In this work we propose a machine learning regression approach for genome-to-phenotype prediction to improve the use of quantitative phenotypes as target variables in functional genomics. We consider first a standard Support Vector Regression (SVR) algorithm and then the L1L2 regression [14] approach. The machine learning methods are part of a software pipeline that implements a complete DAP for regression on genotype data. The L1L2 pipeline also includes a model selection module based on the concept of stability of ranked lists, previously developed for genomic profiling [15]. A procedure testing for markers highly correlated and proximal on the chromosome, termed saturation, is also provided in the pipeline. We present examples of prediction of quantitative phenotypes on a genomewide dataset of 12K SNPs. The dataset used in this study , which we will refer to as the ”GSCAN dataset”, is publicy available (website: http://gscan.well.ox.ac.uk), courtesy of the Wellcome Trust Center for Human Genetics. Data include familiar, genotype and phenotype information from a population of 4 generations of heterogeneous stock mice [16]. Two quantitative phenotypes were used: the percentage of CD8+ cells (CD8+), and the Mean Cell Haemoglobin (MCH). The number of samples is 1521 for %CD8+ and 1591 for MCH. The results from our methods are compared with those of a Reversible Jump Monte Carlo Markov Chain (MCMC) model adapted to fitting quantitative phenotypes and applied to the same dataset [1].

## Results and discussion

**Figure 1:**
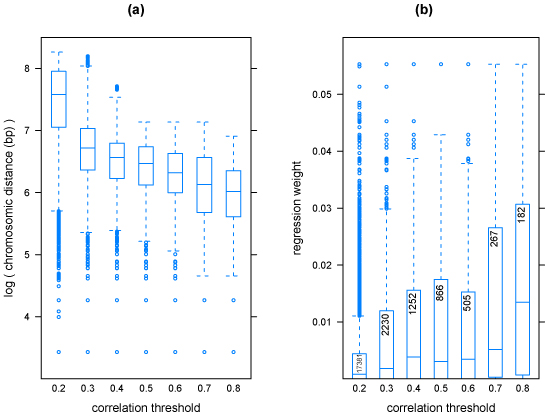
**Distance and regression weights for top-correlated SNPs** For each top-ranked SNP, a set of corresponding top-correlated SNPs at a given correlation threshold is identified. All the chromosome distances from reference top-ranked SNP, and all regression weights are pooled together across all top-ranked SNPs. Numbers inside boxplots indicate the number of top-correlated SNPs; the number of top-ranked SNPs is 51 for the CD8 phenotype shown here. (a) Distributions of chromosome distances between top-ranked and top-correlated SNPs (bp, natural log scale). (b) Distribution of L1L2 regression weights for top-correlated SNPs. Average weight of top-ranked SNPs is 0.07.

**Figure 2:**
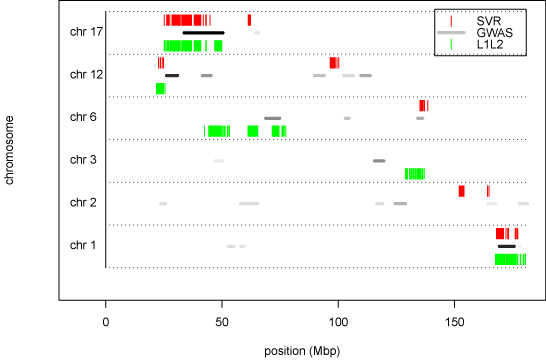
**Top-ranked and top-correlated SNPs for the CD8+ phenotype** SNPs selected for CD8+ phenotype by SVR (*red*), L1L2 (*green*) and GWAS [16] (horizontal segments, levels of gray indicates probability of association;* black:* probability 1,* white:* probability 0). For SVR and L1L2, top-ranked SNPs and the corresponding top-correlated SNPs are shown.

**Figure 3:**
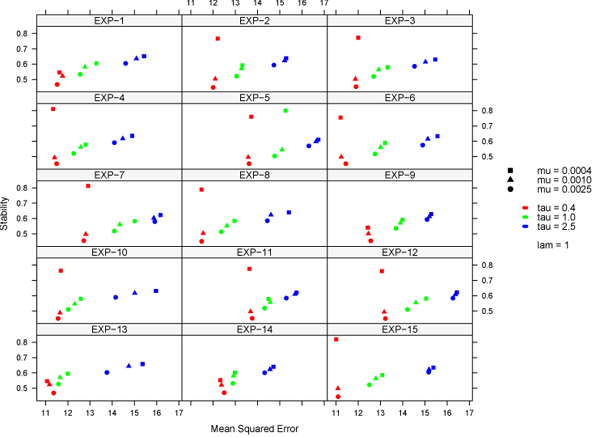
**Accuracy-stability plot for model selection** Accuracy-stability plot for the CD8+ phenotype for 15 development / validation splits. The measure for accuracy is the mean squared error between predicted and actual value of the phenotype, averaged over the 10 Cross-Validations; the measure for stability is the Canberra complete distance for partial lists [15].

For comparability with the reference study [1], the accuracy was measured as the squared correlation coefficient between the predicted phenotype and its actual value on test data, according to an appropriate DAP. The DAP for SVR was chosen so to replicate the one used in [1]. Since this DAP is prone to introduce selection bias (see section Methods), we chose a stricter DAP for L1L2, thus limiting the overestimation of its predictive performance. The learning pipeline was first applied to a dataset where phenotype values had been randomly shuffled in order to check for predictions based on random associations. The prediction accuracy resulted close to zero for SVR and L1L2 regression methods, as expected. Predicted accuracy values for the complete experiment are listed in Tab. 1. Squared correlation coefficients for the three methods were averaged over 15 re-samplings of the development/validation splits. The results shown for SVR have been obtained with a Gaussian kernel (*σ* = 2.5 ⋅ 10^−4^), and figures for the reference MCMC method are reproduced from [1]. For MCH, Table 1 shows an average performance indicator of 0.147 against 0.111 of MCMC, corresponding to an increase of 32%. The increase is statistically significant with respect to both MCMC and L1L2: a p-value < 0.01 was obtained using a t-test (null hypothesis: average values for SVR is less than or equal to that of the other method; a one-sample t-test was used for comparison with MCMC and a Welch two-sample t-test for comparison with L1L2). For CD8+, the same p-values were respectively 0.59 and 0.57: therefore, the difference in the average performances is not statistically significant, i.e. the three methods are equivalent on this phenotype. For each experiment, the L1L2 pipeline yields lists of features ranked by the regression weights. Unlike L1L2, the Gaussian kernel SVR is unable to provide lists of ranked feature: therefore, a linear SVR was used to select features with this approach. The predictive accuracy of the linear SVR was significantly lower than all three reported methods on both phenotypes (data not shown). For both L1L2 and linear SVR, we term ’top-ranked’ the SNPs in the top 10-percentile of the distribution of the weights for at least 14 of 15 experiments. These SNPs are systematically and significantly associated to the given trait. However, correlation between features is characteristic of high throughput molecular data and it is well-known that correlated, functionally important variables may be discarded or poorly ranked. The process of recovering these additional variables is termed saturation. We thus introduce the notion of ’top-correlated’ markers, i.e. SNPs whose population profiles are highly correlated (absolute value of correlation coefficient above a given threshold) with those of top-ranked SNPs. Table 2 shows the characteristics of top-correlated SNPs with respect to their relative top-ranked SNP. Only top-ranked SNPs with at least 5 top-correlated SNPs are shown. The distributions of the chromosome distance and of the regression weights of top-correlated SNPs were analyzed for several values of correlation thresholds (Fig. 1). Top-correlated SNPs are clustered around the reference top-ranked SNPs (Fig. 1a) and as the correlation threshold increases the median of the distribution of distances decreases quickly. The addition of top-correlated SNPs as candidate features for the regression model provides a saturation strategy that may help defining non-punctual loci of interest on the chromosomes. Fig. 1b shows that top-correlated SNPs are generally assigned higher regression weights from the L1L2 algorithm, although some of them are eliminated (regression weight = 0). Thus the impact of the saturation procedure is expected to be limited on the regression model, while yielding a more dense set of candidate markers. The approach has been compared on the GSCAN dataset with a previous GWAS [16]. In Fig. 2 we pool top-ranked and top-correlated SNPs (correlation threshold: 0.8) for the CD8+ phenotype and both methods, and show their position on the genome against GWAS candidate loci [16]. Loci selected by both SVR and L1L2 overlap on most of those selected by the GWAS. Stability of features is crucial for reproducibility and identification of the most relevant biomarkers. The accuracy-stability diagnostic plot in Fig. 3 for the L1L2 method and CD8+ phenotype shows that the same parameter set is optimal on each of the 15 runs (average on the 10 Cross Validations for 9 different parameter sets).

**Table 1:** Prediction accuracy on GSCAN mice data

Method	CD8+	MCH
SVR	0.306 (0.280-0.333)	0.147 (0.125-0.169)
L1L2	0.316 (0.283-0.347)	0.106 (0.095-0.116)
MCMC	0.314	0.111

**Figure 4:**
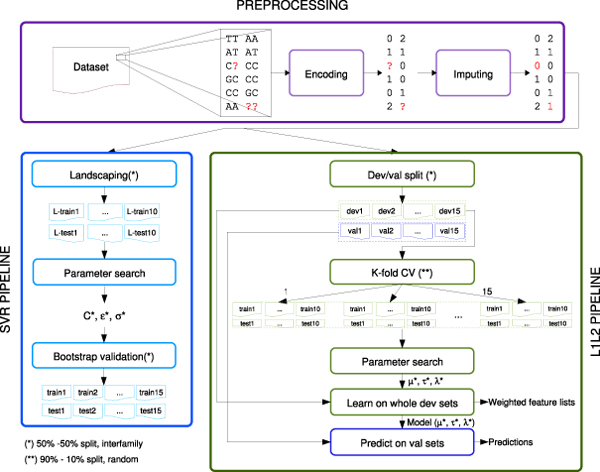
Data Analysis Protocols for the machine learning methods

The squared correlation coefficient between predicted and true values is computed from the pipeline in Fig. 4 by different methods, with 95% bootstrap confidence interval in brackets. MCMC results are reproduced from [1].

**Table 2:** Top-correlated SNPs characteristics

SNP name	chromosome	n	mean distance (bp)	min distance	max distance
mhcCD8a2	6	16	724067.5	2701	1735204
rs13478736	6	13	1372896.5	187157	4078235
mCV24938952	8	12	2824059.5	191536	6126682
rs6375522	1	9	952187.1	115037	2360403
rs13482427	15	9	773579.3	244547	1555507
rs3145663	17	8	1184576.6	293271	2766351
rs3672987	17	7	3346835.3	1194418	5686326
rs13476229	1	6	813948.7	328290	1088152
rs3684143	17	5	292337.2	116575	505713
rs3678696	17	5	452020.4	77709	742231

*n*: number of corresponding top-correlated SNPs; Correlation threshold: 0.8; 5 top-ranked SNPs with no top-correlated SNPs.

## Conclusions

Prediction of quantitative phenotypes from high-throughput genotype data is an emerging research goal with significant applications. It can be envisioned that this predictive modeling problem will evolve into fitting a multidimensional phenotype pattern or a phenotype trajectory. More sophisticated predictive tools still need to be developed to achieve this goal, but it is anyway urgent to deploy experimental setups that can appropriately support model selection and biomarker identification. Here we introduced a framework for the systematic use of machine learning regression methods on whole-genome datasets. Building on results from the FDA’s led MAQC-II initiative, the framework includes a pipeline of procedures (defined through a DAP) to avoid selection bias and ensure reproducibility. The application of the pipeline to up to 550 000 features was made feasible by an efficient software implementation, also suitable for high performance computing facilities. A DAP reproducing those of the original study [1] and employing a Gaussian kernel SVR obtained results comparable with the MCMC method. However the model selection solution does not protect from overfitting and cannot directly derive a list of selected SNP. The L1L2 method was as accurate as the reference study despite the use of a more stringent DAP, and it is able to provide an embedded feature selection which has shown to cope well with the problem of recovering correlated variables. An adjuvant therapy to the issue of correlated variables was proposed with the SNP saturation procedure, based on the concept of top-correlated features. The saturation procedure can be seen as a black box algorithm within the pipeline that automates an analysis by Linkage Disequilibrium after one biomarker is found. SNP saturation also reduces spatial sparsity, because the additional markers are in general close to the top-ranked markers, as shown on the GSCAN data. The finding opens the possibility of encoding by special kernels feature and spatial correlation together.

The L1L2 pipeline also makes use of a model selection criterium aimed at increasing the stability of the list of candidate markers. As a result, the features selected by L1L2 are compatible with results of a previous GWAS on the same dataset [16]. This study confirms that machine learning approaches may support a more effective and reproducible use of multivariate genotype data for the prediction of quantitative traits [17].

## Methods

### Machine Learning methods

To fit quantitative phenotypes from genotypes, a classic LIBSVM implementation [18] of *ε*-SVR [19] was considered in the software solution available on MLOSS (http://www.mloss.org); a Gaussian kernel was used for predictions, and regression weights computed with a linear kernel to rank features. The SVR served as a baseline for comparison to L1L2 [14], a regularization method that outputs the optimal weight vector of a linear regression while maintaining a high sparsity of the solution. It is thus both a regression and a feature selection method. The L1L2 method is an alternative to the elastic net proposed by Zou & Hastie [20] where the Least Absolute Shrinkage and Selection Operator (LASSO) regression [21] is combined with ridge regression. The convex problem for both L1L2 and the elastic net is given by:

	(1)

The solution (*naïve elastic net*) correctly selects the relevant features, but with biased weights. Zou & Hastie [20] corrected by rescaling the weights. In the approach proposed in [14] and used in this study, the correction is done by a Regularized Least Squares (RLS) regression performed only on the subset of features selected after the optimization of w in equation 1. The optimal weights in the RLS regression are found as

	(2)

where  and  refer to the input data and the regression weights restricted to the subset of selected features. Thus, *µ* and *τ* modulate the feature selection, whereas the regularization parameter λ of the RLS controls the weight bias. The minimization of Eq. 1 is computed with a modified gradient descent algorithm, which makes use of weight values shrinkage through an iterative thresholding algorithm. For a more detailed description of the method, we refer the reader to the original paper [14]. L1L2 has been applied to define a transcriptomic profile of hypoxia in neuroblastoma cell lines from classification through regression [22]. A type of L1L2 was also used on eQTL datasets to predict the regulatory potential [23]. The L1L2 regression used in this paper is efficiently implemented through functions from the Open Source mlpy package (also available on MLOSS).

### Data Analysis Protocols

We adopted two DAPs (workflow displayed in Fig. 4), composed by a common preprocessing step (Fig. 4, top), for genotype encoding and imputation of missing values, and method-specific model selection sections (Fig. 4, bottom left: SVR, bottom right: L1L2).

#### *Preprocessing*

Genotype data are generally encoded with {0, 1, 2} (dominant homozygous, heterozygous, recessive homozygous respectively): this representation has the biological meaning of the number of allelic deviations of the SNP from the dominant homozygous. However, different representations may yield different results. We thus preliminarily compared the proposed encoding with a {−1, 0, 1} encoding, a binary {100, 010, 001}, and one based on the relative frequency of each allelic class for given SNP over the total sample population. For SVR, no significant difference in predictive power was detected between the {0,1,2} and the {−1,0,1} encoding; slightly worse performances were obtained with the frequency-based encoding and significantly worse with the binary {100, 010, 001} encoding. Therefore, we kept to the standard {0,1,2} encoding. Recently, Liu et al. [24] have proposed a sparse binary encoding {00, 01, 11} for a bioinformatics application to ancestry inference. This encoding will be tested in future applications of our regression framework. Finally, missing data (SNPs that are not called) were randomly imputed with probability equal to the relative frequency of each allele at that locus in the population. Random imputation is not biologically plausible, as it will introduce Mendelian errors in the samples and does not take into account linkage disequilibrium effects. More accurate imputation methods for uncalled SNPs have been proposed and are reviewed in [25]. However, the proportion of missing data in the considered dataset is small: 0.14% of all data points are uncalled, with a maximum uncalled samples per SNP of 5.2%. Only 11 SNPs of over 12,000 had more than 2% uncalled samples and only 116 more than 1%. Thus, we expect that the errors introduced by the random imputation will hardly impact the predictive ability of the algorithms.

#### *Model selection*

For SVR (Fig. 4, bottom left) we adopted a model selection scheme replicating that of the reference study [1] for comparability. Optimal parameters were found by grid search on 50% bootstrap (10 replicates). The model was selected based on the maximal mean squared correlation coefficient between the predicted and actual output, and then validated on 15 train/test bootstrap. Members of a family in GSCAN were all assigned to either the training or the test set (interfamily sampling), thus avoiding information leakage due to very high genetic similarity between individuals in the same family. This DAP introduces bias in the evaluation of the method’s accuracy, since it uses in the validation stage the same information (samples) already exploited in model selection: therefore, its results are potentially over optimistic. For this reason, we used a more sophisticated protocol for L1L2 (Fig. 4, bottom right), from the guidelines of the MAQC-II project [11]. The only modification that we introduce to the MAQC-II protocol in the regression context is the model selection stage in the accuracy-stability space, required to choose the optimal L1L2 parameter triple. Given the 15 development/validation interfamily splits, model selection in the accuracy-stability space [15] was obtained by internal 10-fold Cross Validation on each development dataset. For each of the 15 experiments, we compute the mean squared error between predicted and true value as a measure of accuracy, and the stability of marker lists through the Canberra distance indicator [15]. For two ranked partial lists *L*_1_, *L*_2_ of length respectively *p*_1_ and *p*_2_ on a common set of *p* features *F*, their Canberra distance Ca(*L*_1_, *L*_2_) is defined as follows: 

where *S_p_* is the symmetric group on *p* symbols, and *τ_i_* is the permutation of *S_p_* corresponding to *L_i_* (*i* = 1,2) for a given order of the features* F* and . For a given set* L* of partial lists, the Canberra stability indicator is defined as the mean of all the mutual Canberra distances among the elements of L: the choice of the mean is justified by Hoeffding’s theorem on the asymptotic normality of the distribution of Canberra distances. The Canberra distance was chosen as the (dis)similarity measure because of its intrinsic larger penalization of changes of rank in the top position of the ranked lists. For a complete mathematical description and a few application examples see [26,27]. The model defined by the optimal (*µ*, *τ*, λ) in terms of maximal accuracy and marker list stability was then trained and evaluated on each development/validation split. The L1L2 algorithm and its protocol are implemented within the mlpy Python package and run on Kore, the FBK Linux High Performance Computing facility. It was successfully tested on up to 550k features and a few thousands of samples.

## List of abbreviations used

DAP: Data Analysis Protocol; GWAS: Genome-Wide Association Study; LASSO: Least Absolute Shrinkage and Selection Operator; MCH: Mean Cell Haemoglobin; MCMC: Monte Carlo Markov Chain; RLS: Regularized Least Squares; SNP: Single Nucleotide Polymorphism; SVM: Support Vector Machine; SVR: Support Vector Regression.

## Competing interests

The authors declare that they have no competing interests.

## Authors' contributions

GG participated in the design of methods, performed the experimental validation and drafted the manuscript. GJ participated in the machine learning method design and revised the manuscript. CF designed the study, analyzed results and drafted the manuscript. All authors read and approved the final manuscript.
